# An IoT-Enabled Wearable Device for Fetal Movement Detection Using Accelerometer and Gyroscope Sensors

**DOI:** 10.3390/s25051552

**Published:** 2025-03-02

**Authors:** Atcharawan Rattanasak, Talit Jumphoo, Wongsathon Pathonsuwan, Kasidit Kokkhunthod, Khwanjit Orkweha, Khomdet Phapatanaburi, Pattama Tongdee, Porntip Nimkuntod, Monthippa Uthansakul, Peerapong Uthansakul

**Affiliations:** 1 School of Telecommunication Engineering, Suranaree University of Technology, Nakhon Ratchasima 30000, Thailand; 2 Institute of Research and Development, Suranaree University of Technology, Nakhon Ratchasima 30000, Thailand; jumphoo@sut.ac.th (T.J.);; 3 Department of Integrated Engineering, Rajamangala University of Technology Tawan-Ok, Chanthaburi 22210, Thailand; 4 Department of Telecommunication Engineering, Faculty of Engineering and Technology, Rajamangala University of Technology Isan (RMUTI), Nakhon Ratchasima 30000, Thailand; 5 School of Obstetrics and Gynecology, Institute of Medicine, Suranaree University of Technology, Nakhon Ratchasima 30000, Thailand; 6 School of Medicine, Institute of Medicine, Suranaree University of Technology, Nakhon Ratchasima 30000, Thailand

**Keywords:** fetal movement detection, internet of things, wearable device, machine learning

## Abstract

Counting fetal movements is essential for assessing fetal health, but manually recording these movements can be challenging and inconvenient for pregnant women. This study presents a wearable device designed to detect fetal movements across various settings, both within and outside medical facilities. The device integrates accelerometer and gyroscope sensors with Internet of Things (IoT) technology to accurately differentiate between fetal and non-fetal movements. Data were collected from 35 pregnant women at Suranaree University of Technology (SUT) Hospital. This study evaluated ten signal extraction methods, six machine learning algorithms, and four feature selection techniques to enhance classification performance. The device utilized Particle Swarm Optimization (PSO) for feature selection and Extreme Gradient Boosting (XGB) with PSO hyper-tuning. It achieved a sensitivity of 90.00%, precision of 87.46%, and an F1-score of 88.56%, reflecting commendable results. The IoT-enabled technology facilitated continuous monitoring with an average latency of 423.6 ms. It ensured complete data integrity and successful transmission, with the capability to operate continuously for up to 48 h on a single charge. The findings substantiate the efficacy of the proposed approach in detecting fetal movements, thereby demonstrating a practical and valuable technology for fetal movement detection applications.

## 1. Introduction

### 1.1. Background and Motivation

Maintaining good health for both the fetus and mother during pregnancy is the primary goal of obstetric healthcare [1]. Many countries are facing challenges in their population due to decreased birth rates, infertility, and fetal mortality [2]. These issues show how crucial every pregnancy is for maintaining population growth and fulfilling the needs of a family. A decrease in the birth rate further results in an increasing old-age population, which burdens the healthcare system [3]. Fetal movement is an important biological indicator of fetal well-being as it reflects the maturity of the central nervous system, muscles, and bones. A significant decrease or cessation of fetal movements often precedes fetal death in utero from chronic causes like toxemia, even when heartbeats remain detectable [4]. Reduced fetal activity could be a sign of severe conditions like ineffective placental function, abnormality in the uterus, and even decreased growth of the fetus, which could end up in intrauterine fetal death if not treated at the right time [5]. Continuous monitoring of fetal movements has the potential to warn of fetal compromise and aid clinical decision-making and ultimately prevent fetal death [6].

Traditional techniques for the assessment of fetal movement include intermittent auscultation (IA), ultrasound examinations, and external fetal monitoring (EFM), which typically require healthcare facilities with adequate infrastructure [7]. While IA is popular for its simplicity and convenience, it relies heavily on accurate listening skills [8]. EFM provides detailed pregnancy information but demands familiarity, skill, and extensive experience for accurate interpretation [9]. However, as pregnant women are a vulnerable group susceptible to infection, and the risk of healthcare-associated infections continues to rise, promoting home-based self-monitoring for pregnant women has become increasingly important to reduce associated infection risks [10]. A study published in 2020 highlighted that 63.4% of pregnant women worried about contracting infections in hospitals, yet they also feared delays in fetal health checks if they did not visit the hospital [11]. For home monitoring, after 20 weeks of pregnancy, doctors typically ask pregnant women to spend one hour after meals each day monitoring and recording fetal movements, keeping these records for medical review [12]. While this method is suitable for self-monitoring, some studies indicate that maternal perception sensitivity varies considerably and can be influenced by external environmental factors, leading to high variability [13]. Additionally, self-monitoring through movement counting can cause distress and anxiety as mothers must constantly focus on counting fetal movements [14]. The limitations of traditional and home-based monitoring methods have led to the development of innovative prenatal care technology beyond simple remote consultations [15]. The integration of Internet of Things (IoT) technology enhances the communication between healthcare providers and pregnant women, enabling timely interventions when needed [16].

The rapid advancement of IoT-enabled wearable devices has significantly influenced modern healthcare, enabling continuous and real-time physiological monitoring through smart sensors and cloud-based analytics. Wearable technologies are now widely utilized for heart rate monitoring, blood oxygen measurement, activity tracking, and sleep analysis, forming the foundation for their integration into prenatal care [17]. The Internet of Medical Things (IoMT) has further expanded healthcare applications by incorporating AI-driven disease prediction, remote biomarker detection, and blockchain-secured health records, allowing seamless, real-time data transmission from wearable sensors to cloud platforms [18]. A cardio-specific IoT-based monitoring system has demonstrated the potential of integrating ECG, PPG, and motion sensors to assess Heart Rate Variability (HRV) and detect arrhythmias, showcasing the feasibility of such technology in maternal–fetal health tracking [19]. Additionally, cloud-based health-monitoring platforms, including Google Fit, Apple Health, and Samsung Health, have enhanced accessibility to wearable health data, supporting healthcare providers in delivering remote and personalized care [20]. Given these advancements, IoT-based wearable systems can revolutionize prenatal care by enabling non-invasive maternal and fetal health monitoring, reducing dependency on traditional hospital-based checkups while enhancing early detection and intervention capabilities [21]. With these advancements, researchers have explored various IoT-based wearable systems for prenatal care, demonstrating their feasibility in real-world applications. For instance, an automated system using wireless electrohysterography (EHG) sensors and smartphones has been developed to detect uterine contraction patterns, providing early warnings of premature labor [22]. Another study tested the feasibility of smartwatches for monitoring step counts, sleep, and heart rate over a 7-month period, indicating their potential for long-term maternal health tracking [23]. Further research has integrated smartwatch data with mobile applications, enabling the continuous monitoring of stress, sleep quality, and daily physical activity [24]. Meanwhile, advancements in IoT architecture have facilitated the integration of various wearable sensors, including heart rate monitors, motion sensors, temperature sensors, blood pressure monitors, and oxygen saturation sensors, broadening the scope of maternal–fetal health applications [25]. Recent developments have also led to the creation of an IoT-based labor progress monitoring system, utilizing force sensors to track uterine contractions and AI algorithms to analyze data, ultimately assisting in hospital admission timing recommendations [26]. While IoT applications in prenatal care have expanded rapidly, research focusing on fetal movement detection has shown promising progress. Notably, Qin et al. [27] developed a wearable system using two acceleration sensors with a Cortex-M4 control unit, transmitting data via Bluetooth to a smartphone application. Similarly, Zhao et al. integrated accelerometer sensors with machine learning to create an automated fetal movement monitoring system, highlighting the potential of AI-enhanced IoT solutions in prenatal healthcare [28].

### 1.2. Related Studies

Accurate detection of fetal movement has become a critical focus in prenatal care, as it serves as an essential indicator of fetal well-being. This section reviews the latest methodologies employed in fetal movement detection, focusing on algorithms, signal processing techniques, and sensor integration. The objective is to identify technological gaps that could serve as a foundation for future research.

Mesbah et al. [29] developed an accelerometer-based fetal activity monitor (AFAM) using four tri-axial accelerometers (three on abdomen, one on chest as reference). Their method computed root mean square (RMS) of acceleration magnitude from three axes. Movement was classified as fetal when RMS exceeded the median value. In tests with three mothers carrying 35-week fetuses, the system showed 76% sensitivity (SEN) and 55% specificity (SPEC), outperforming maternal perception, which achieved only 36% SEN. While SPEC remained low due to maternal movement interference, detection improved with gestational age due to stronger fetal movements.

Altini et al. presented significant developments in fetal kick detection through two research works. In their first study [30], they integrated data from six accelerometers (five on the abdomen and one on the back as a reference), utilizing basic time-domain features such as mean, standard deviation (SD), interquartile range, and correlations between axes as well as with the reference sensor. The classification using Random Forest (RF) achieved 75% SEN and 65% PPV. Subsequently, in [31], they developed a novel approach that significantly differed from previous works by proposing a single device that combines accelerometer and electromyography (EMG) sensors. They introduced variable-length feature technique, which analyzes data through both short and long time windows to distinguish between fetal and maternal movements. This innovation substantially reduced system complexity while maintaining performance, achieving 76% PPV and 64% SEN, comparable to multi-sensor systems.

Abeywardhana et al. [32] presented a fetal movement detection system using a tri-axial accelerometer. Their approach focused on distinguishing fetal movements from maternal respiratory movements and laughter using auto-correlation matrix (ACM) analysis. The method employed eigenvalue and eigenvector analysis combined with a modified biased surface separation technique, achieving 95% Accuracy (ACC) in fetal movement detection. This simple approach required minimal training data and effectively reduced misclassification of respiratory movements.

Zhao et al. [28] proposed a fetal movement detection algorithm using accelerometer signals. Their method employed IIR band-pass filtering and cross-correlation for noise and maternal movement reduction, followed by a fuzzy Adaptive Resonance Theory Mapping (ARTMAP) classifier for movement classification. While this approach enabled incremental learning without complete retraining, no performance metrics were reported to evaluate the system’s effectiveness.

Bobrova et al. [33] proposed an algorithm for fetal activity monitoring using accelerometer data from the maternal abdomen. Their method employed band-pass filtering to remove high-frequency noise, slow respiratory waves, and maternal heartbeat artifacts, then applied a fixed threshold to identify peaks corresponding to fetal movements. In the same year, they also introduced a passive fetal activity registration approach, exploring accelerometers, gyroscopes, and acoustic sensors to enhance the early detection of critical fetal conditions, along with modeling the signal source and justifying the key parameters used in developing that model [34].

Somathilake et al. [35] developed a fetal movement monitoring system using a single accelerometer. Their signal processing pipeline involved Wiener filtering and wavelet transform for feature extraction. They evaluated two deep learning (DL) approaches for movement classification: CNN and GRU-based RNN. In experiments with 13 pregnant women, the CNN achieved 75% ACC on the test set, while the GRU-based RNN showed superior generalization to new subjects, though inter-subject variability in fetal movement patterns remained a key challenge.

Xu et al. [36] developed a wearable fetal movement detection system using two accelerometers. For signal processing, they employed wavelet denoising and extracted 78 features comprising statistical, morphological, and wavelet features, with the Synthetic Minority Over-sampling Technique (SMOTE) used to address class imbalance before training. Among nine compared classifiers, the Extra Trees Classifier achieved the best performance with 82.40% SEN, 86.10% precision (PRE), and 84.20% F1-score (F1). In experiments with 20 pregnant women using ultrasound as ground truth, using two accelerometers improved detection ACC by 10% compared to a single sensor.

Ouypornkochagorn et al. [37] proposed a fetal movement detection system using acoustic signals attached to the maternal abdomen. They transformed the signals into spectrograms to extract up to 1680 features and compared four different classifiers, finding that the Convolutional Neural Network (CNN) achieved the highest accuracy of 94.9% and an F1-score of 95.2%. Subsequently, they developed a portable fetal movement detection system for home use [38], tested under conditions that allowed mothers to move freely, and compared the same four classifiers. The CNN again exhibited the best performance, with an F1-score of 81.6%.

Qin et al. [27] proposed a wearable fetal movement detection system using two accelerometers. Their signal processing employed a threshold-based approach consisting of Kalman filtering for noise reduction, followed by amplitude thresholding to distinguish fetal from maternal movements, and Orthogonal Matching Pursuit (OMP) algorithm for classification. Testing with four pregnant volunteers showed that both recognition rate and PRE were equal to 89.74%.

Ghosh et al. [39] first explored a multi-modal wearable system for fetal movement detection by integrating accelerometers, acoustic sensors, and piezoelectric diaphragms into a single belt. They employed a fusion of data-dependent thresholding and a machine learning approach to mitigate maternal body movement artifacts. Tested in a home-based scenario with five pregnant women, the system achieved an F1-score of 79% using a Neural Network (NN) classifier, demonstrating the benefits of heterogeneous sensor fusion. Building upon this, Ghosh et al. [40] conducted a comparative study of the individual sensors. Their signal processing pipeline included Hann windowing and Welch’s method for Power Spectral Density (PSD) estimation. Features were extracted in both time and frequency domains using FFT, then combined through binary maps and logical OR operations before classification with an NN using cross-validation. Testing with the same cohort showed that the piezoelectric sensor achieved an F1-score of 71%, while both the accelerometer and acoustic sensor scored 65%. Combining all three improved the F1-score to 79%, further reinforcing the advantage of sensor fusion for fetal movement detection.

The reviewed studies highlight the evolution of fetal movement detection systems, from basic sensor applications to more sophisticated integrated solutions. Accelerometer sensors remain the most popular technology for detecting fetal movements [27,28,29,30,31,32,35,36,39,40] as they can directly detect vibrations from fetal movements. However, to enhance detection efficiency, several studies have developed approaches that integrate various types of sensors, such as acoustic sensors [37,38], piezoelectric diaphragms [40], and EMG sensors [31]. In terms of practical implementation, previous research has developed wearable devices that connect to IoT systems and transmit data to the cloud for further analysis [27], enabling doctors to monitor data remotely through smartphone applications. Subsequently, ref. [28] introduced AI-embedded techniques that directly embed AI models in microcontroller (MCU) devices for on-device processing, rather than relying solely on cloud processing. Along with sensor development, advancements in signal processing and ML continue to progress, with applications of DL techniques through CNNs and GRU-based NNs, alongside advanced signal processing methods such as wavelet transform and spectrogram analysis, to improve overall system performance [35,37,38].

Despite the promising results of these advanced algorithms on high-performance computers, deploying them on resource-constrained MCU devices remains challenging. This study approach focuses on developing efficient traditional ML algorithms suitable for MCU implementation while maintaining competitive performance. To enhance the effectiveness of our MCU-based solution, we propose two key innovations. First, we integrate gyroscope sensors with accelerometers to improve detection efficiency, as gyroscopes can detect rotational movement that helps distinguish between maternal and fetal movements more clearly. Second, we employ various optimization techniques such as Particle Swarm Optimization (PSO), Genetic Algorithm (GA), Firefly Algorithm (FA), and Whale Optimization Algorithm (WOA) for feature selection and hyperparameter optimization to improve model performance. Through these approaches, we aim to develop an effective MCU-based system that supports remote medical access through cloud platforms, facilitating telemedicine and enabling the convenient monitoring of fetal abnormalities from home.

**Figure 1:**
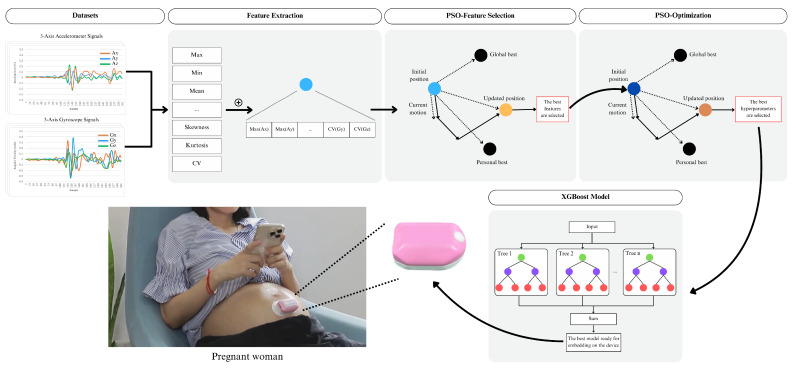
Overview of the proposed fetal movement monitoring system framework.

Table 1 shows the evolution of fetal movement detection systems. Early systems [29,30,32,37] used non-wearable accelerometer sensors. Subsequently, the systems evolved into wearable devices [27,28,31,35,36,38,40]. There has also been development in sensor integration, including EMG [31], acoustic sensors [37,38], and piezoelectric sensors [40]. During 2019–2023, IoT technology was introduced [27,28] but only one study [28] combined IoT with AI-embedded and remote medical access features. Building upon these technological advancements, our research introduces an innovative fetal movement monitoring system that integrates wearable technology embedded with ML to enhance detection performance. A lightweight, non-invasive device captures real-time accelerometer and gyroscope signals, with feature extraction and PSO enabling optimal feature selection and model tuning. Figure 1 illustrates our proposed framework, highlighting the seamless integration of data acquisition, processing, and classification. The developed system delivers the following key contributions:

1. This study introduces a novel sensor fusion approach by combining accelerometer and gyroscope data to improve the performance of fetal movement detection. PSO algorithm is utilized for feature selection, enabling the selection of optimal features from both sensors. Additionally, PSO is used for hyperparameter tuning to determine the best parameters, further improving detection performance.
2. Unlike existing methods that rely on cloud processing [27] or complex Deep Neural Networks (DNNs) [35,37,38], this work implements a lightweight ML classifier on an ESP-32 microcontroller. While ref. [28] also uses an AI-embedded system, the authors do not report key performance metrics such as SEN, PRE, or F1. Our lightweight ML model achieves superior performance, enabling continuous monitoring both within and outside hospital settings.
3. This study integrates accelerometer and gyroscope data with IoT connectivity to facilitate remote access and medical oversight. The collected data are transmitted to a cloud-based platform, enabling healthcare providers to review fetal movement information and thereby support telemedicine practices.
4. The proposed system was developed and validated using data collected from 35 pregnant women at Suranaree University of Technology (SUT) Hospital, representing the largest participant group in fetal movement detection studies to date. Preliminary results show that the system achieved 90.00% SEN, 87.46% PRE, and an F1 of 88.56%. The system also guarantees 100% data integrity and supports continuous operation for up to 48 h on a single charge with an average transmission latency of 423.6 ms.

**Table 1:** Comparison of existing detection systems.

Reference	Sensor	Wearable Device	Participant	IoT System	AI -Embedded	Medical Remote Access
Mesbah et al. (2011) [29]	Accelerometer	×	3	×	×	×
Altini et al. (2016) [30]	Accelerometer	×	6	×	×	×
Altini et al. (2017) [31]	Accelerometer, EMG	✓	22	×	×	×
Abeywardhana et al. (2018) [32]	Accelerometer	×	N/A	×	×	×
Zhao et al. (2019) [28]	Accelerometer	✓	14	✓	✓	✓
Somathilake et al. (2022) [35]	Accelerometer	✓	13	×	×	×
Xu et al. (2022) [36]	Accelerometer	✓	20	×	×	×
Ouypornkochagorn et al. (2023) [37]	Acoustic	×	12	×	×	×
Qin et al. (2023) [27]	Accelerometer	✓	4	✓	×	×
Ghosh et al. (2024) [39]	Accelerometer, Acoustic, Piezoelectric	✓	5	×	×	×
Ghosh et al. (2024) [40]	Accelerometer, Acoustic, Piezoelectric	✓	14	×	×	×
Ouypornkochagorn et al. (2025) [38]	Acoustic	✓	7	×	×	×
**Proposed**	**Accelerometer, Gyroscope**	✓	**35**	✓	✓	✓

## 2. Materials and Methods

### 2.1. Design and Implementation of Fetal Detection Device

#### 2.1.1. Device Design

**Figure 2:**
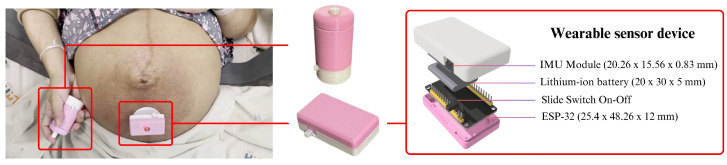
Overview of the wearable fetal movement monitoring system components.

The wearable prototype is shown in Figure 2. The core component of the device is a single IMU module, with a size of 25.90 × 15.50 mm, which combines a three-axis accelerometer and a three-axis gyroscope that had measuring ranges of ±16 g and ±2000 °/s, respectively. The sensor had a 16-bit resolution and a maximum sampling rate of 190 Hz for both acceleration and angular velocity. The processing unit of the device is the ESP-32 microcontroller, which is a powerful and cost-effective platform for developing IoT applications with dimensions of 25.40 × 48.26 mm. It was chosen for its low power consumption and integrated wireless communication features [41]. The ESP-32 features a dual-core Tensilica LX6 microprocessor with clock speeds up to 240 MHz and 520 KB of SRAM. It supports Wi-Fi in the 2.4 GHz band (IEEE 802.11 b/g/n) for stable, high-speed data transmission and includes Bluetooth Low Energy (BLE) for seamless communication with smartphones, enabling real-time monitoring and data logging [42]. Powering the device is a lithium-ion battery with a 20 × 30 mm, which is designed to be rechargeable, providing convenience and reducing the need for frequent replacements, which is essential for a wearable device intended for continuous use. The device incorporates a slide switch for effortless on/off functionality, enabling the user to control it effortlessly. A custom-designed casing encloses the entire device, ensuring safe containment and protection of all components from external damage. The total weight of the device is 25 g. The device was attached to the lower abdomen of all participants using 3M medical-grade adhesive patches. Furthermore, a handheld button device, which operates in two states (0 by default and 1 when pressed), allows the expectant mother to manually record any movements she detects.

#### 2.1.2. Cloud-Based Fetal Movement Monitoring System with Mobile Interface

**Figure 3:**
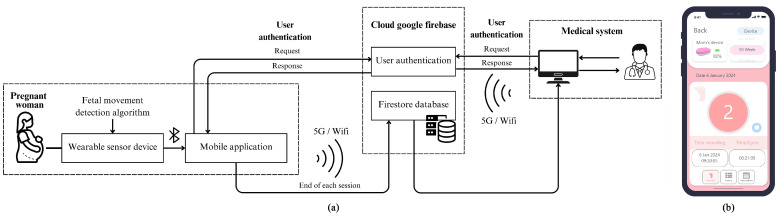
Fetal movement monitoring system with mobile interface (**a**). The system architecture for fetal movement monitoring. (**b**) The user interface of the mobile application designed for pregnant women.

The system architecture integrates a wearable sensor device and a mobile application to detect and monitor fetal movements, as illustrated in Figure 3a. The wearable sensor device, equipped with a fetal movement detection algorithm, is worn by the pregnant woman. This device continuously monitors fetal movements and transmits the data via Bluetooth to the mobile application. The application, developed with flutter, offers a seamless cross-platform experience, ensuring consistent performance on both iOS and android devices [43]. It displays real-time information, including the fetal movement count, which is processed through a detailed decision-making process involving feature extraction and ML classifiers, further elaborated in Section 2.4 and Section 2.5, with performance evaluations discussed in Section 2.6. Additionally, the app displays important information such as the date, time, gestational age, battery percentage, and any symptoms experienced by the mother during the use of the device.

Before accessing the mobile application, users must log in for authentication using the cloud services provided by Google Firebase, ensuring secure access to the system. This process guarantees that only authorized users are able to utilize the features and services of the application, thereby maintaining data integrity and security [44]. Upon successful user authentication, the device automatically begins detecting fetal movements. The data are subsequently stored in the Firestore database at the end of each monitoring session. The system supports both 5G and Wi-Fi for efficient data transfer.

**Figure 4:**
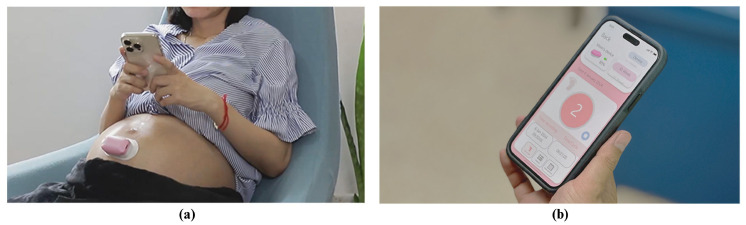
Real-world using wearable fetal movement detection device with mobile application. (**a**) A wearable device on a pregnant woman, connected to a smartphone for fetal monitoring. (**b**) A smartphone displaying real-time fetal movement data.

Medical experts can access the stored data, which also authenticate users via Firebase. This enables doctors to monitor fetal movements remotely, providing timely and informed medical advice without requiring physical hospital visits. The user interface of the mobile application, depicted in Figure 3b, is designed to be intuitive and user-friendly. Furthermore, the real-world use of a wearable fetal movement detection device with a mobile application can be seen in Figure 4.

### 2.2. Participants and Ethical Considerations

This study recruited a total of 35 pregnant women participants from SUT Hospital. The participants had an average age of 28.89 ± 5.48 years. Their average gestational age was also 36.22 ± 2.70 weeks. The average body weight among the women was 73.20 ± 13.60 kg, and their average abdominal circumference measured 102.84 ± 14.35 cm. For demographic information and further details, refer to Table 2.

Ethical considerations were prioritized in this study. This work obtained ethics committee approval from SUT’s Human Research Ethics Committee (License EC-66-33, COA number 31/2566). Participants provided written informed consent after being fully informed about the research objectives, procedures, and their rights to withdraw at any time without consequences. Strict data protection measures were implemented to safeguard participants’ privacy and maintain the integrity of sensitive health information. These measures included data anonymization, secure storage, and controlled access. Participants’ identifiable information was separated from their measurement data and replaced with unique coded identifiers to ensure confidentiality. Access to the data was limited to authorized personnel only, ensuring the privacy of participants throughout the study. These practices underline the ethical commitment of this research to protect the rights and data of all participants involved.

### 2.3. Capture and Labeling of Movement Signals

Before starting the experiments, the expert doctor explained the study’s purpose, duration, and the data collection process in detail. Participants were prepared in a calm and relaxed state, lying comfortably. During the sessions, they were allowed to engage in light activities, such as gentle rolling to the left or right, limb movements, body stretching, breathing, talking, and using mobile phones, to maintain a natural and stress-free environment.

The device was employed to gather the data from a total of 35 participants, with each session lasting approximately 25 min. To facilitate analysis, the dataset was segmented into 5-second windows, following the methodology outlined in the literature [36]. This segmentation allows for detailed examination of movement patterns over short time frames. Given this window size, each participant contributed 300 segments.

Each of the 300 segments was then classified into two categories: fetal movement data and non-fetal movement data. The number of fetal movement data varied among participants during the data collection process, as detailed in Table 2. Data confirmed as fetal movements through dual-labeling were classified as fetal movement signals, while the remaining data, including background noise and body movements, were categorized as non-fetal movement signals.

**Figure 5:**
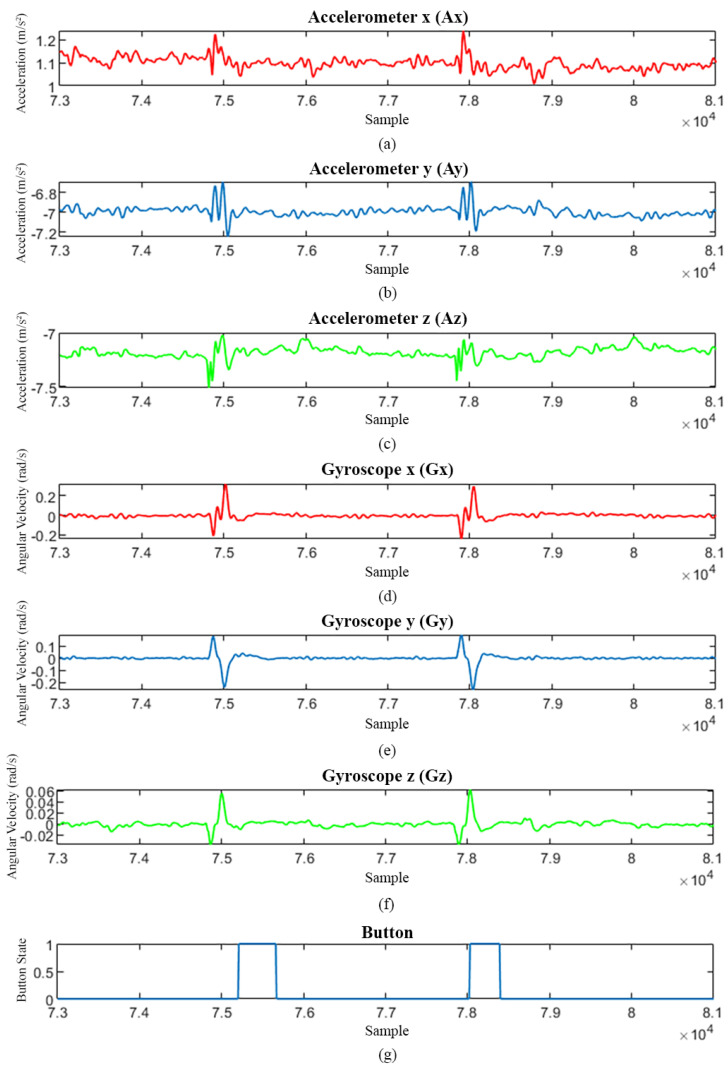
(**a**–**c**) represent the accelerometer signals along the *x*, *y*, and *z* axes, respectively. (**d**–**f**) represent the gyroscope signals along the *x*, *y*, and *z* axes, respectively. (**g**) The signal from the button device, indicating the moments when the mother perceives fetal movements. It has been observed that the perception of fetal movements by the mother lags behind the actual detected fetal movement signals.

In summary, the total data collected from all 35 participants included 100 data for fetal movement signals and 10,150 data for non-fetal movement signals, such as gentle rolling to the left or right, limb movements, body stretching, breathing, talking, and using mobile phones. Examples of characteristics of the raw signals are illustrated in Figure 5.

The collected movement signals were labeled using a two-step process. Initially, signals were labeled by a button press from the mother whenever she felt fetal movement. Then, these labels were reviewed and confirmed by a medical expert from SUT Hospital, providing an additional layer of validation that the signals indeed represented fetal movement. This dual-labeling approach helps ensure the reliability of the dataset used for subsequent analysis and model training.

### 2.4. Feature Extraction and Selection

#### 2.4.1. Feature Extraction

Our wearable device is designed to collect fetal movement signals from the abdominal area. However, these signals are often contaminated with noise from various sources, such as the movements made by pregnant women, talking, and other activities during the tests. As a result, using raw signals for detecting fetal movements is challenging. Therefore, feature extraction and feature selection techniques are crucial for the initial processing of the data, enabling us to distinguish between different signal characteristics effectively [45].

In this study, we extracted common statistical features, as suggested in [46], to characterize different aspects of fetal movements. Maximum (Max) and minimum (Min) values indicate the intensity of strong kicks or movements, while mean, median, and mode help distinguish between fetal and maternal movements. SD measures signal variation patterns characteristic of fetal activity. Additionally, variance (Var), skewness (Skew), kurtosis (Kurt), and coefficient of variation (CV) were extracted to analyze signal distribution and variability. These fundamental statistical descriptors are particularly well suited for ML in fetal movement detection, as they provide a robust and interpretable representation of the signal. These features enable ML algorithms to effectively learn subtle distinctions in the data, ultimately improving classification performance.

The analysis of fetal movement signals involves extracting statistical features from individual axes of accelerometer (*x*, *y*, *z*) and gyroscope (*x*, *y*, *z*) sensor data. Ten distinct statistical measures were computed for each single axis, and with all six axes combined, this yielded a total of sixty features. These extracted features $F_{i,j}$ can be mathematically structured as

(1) $$F=\{F_{i,j}:i\in \left\{1,\cdots ,10\right\},j\in \left\{1,\cdots ,6\right\}\}$$

where *i* (ranging from 1 to 10) represents statistical measures in the following order: Max, Min, mean, median, mode, SD, Var, Skew, Kurt, and CV. The definitions and detailed descriptions of these statistical measures can be found in Table 3. Meanwhile, *j* (ranging from 1 to 6) represents the signal components: Ax,Ay,Az (accelerometer signals along the *x*, *y*, and *z* axes) and Gx,Gy,Gz (gyroscope signals along the *x*, *y*, and *z* axes), respectively. Each feature $F_{i,j}$ is calculated by applying a specific statistical measure (*i*) to a given signal component (*j*). For example, $F_{1,1}$ represents the maximum value of the accelerometer signal along the *x*-axis (${\mathrm{Max}}_{Ax}$).

#### 2.4.2. Feature Selection

Feature selection is a critical step in data preprocessing that aims to improve the performance of classification models by reducing dimensionality and eliminating redundant or irrelevant features [47]. This process not only enhances computational efficiency but also minimizes the risk of overfitting, ensuring that the model focuses exclusively on the most relevant attributes. To address the challenges associated with high-dimensional datasets, this study employs four optimization algorithms: PSO, GA, FA, and WOA. Each algorithm is chosen for its unique strengths in feature selection. PSO is recognized for its ability to rapidly converge to optimal solutions while efficiently exploring the feature space, making it highly suitable for problems requiring quick and effective feature reduction. GA, in contrast, provides exceptional flexibility in handling complex and non-linear optimization problems, allowing it to adapt to diverse datasets and objectives. FA excels at escaping local optima, ensuring that the selected feature set captures global patterns rather than being constrained by suboptimal solutions. Finally, WOA is particularly effective in exploring global search spaces in high-dimensional data, offering robust capabilities for identifying the most informative features in datasets with complex structures. These methods enable a thorough exploration of potential feature subsets, and when their results are compared, they allow for selecting the most suitable feature selection method for the dataset used in this study. The details of each algorithm are as follows:

- **PSO**: A population-based stochastic optimization technique inspired by the social behavior of birds flocking or a school of fish. In PSO, each potential solution, called a particle, represents a subset of features and flies through the search space by following the current optimum particles. Each particle adjusts its position based on its own experience (personal best) and the collective knowledge of the swarm (global best). The objective function was to maximize the F1 of the classification model, as F1 provides a balanced measure between PRE and SEN, which is crucial for fetal movement detection. The fitness function, which evaluates the quality of feature subsets, was defined as the F1 calculated through cross-validation. This technique was used in our study with n_particles = 30, max_iter = 100, c1 = 2, c2 = 2, where c1 and c2 are acceleration coefficients that control the influence of personal and global best solutions. These values were chosen based on standard configurations to balance exploration and exploitation effectively while avoiding premature convergence. During optimization, particles explored different feature combinations to identify those that contribute most significantly to improving classification performance. Details are provided in [48].
- **GA**: An optimization technique based on the principles of natural selection and genetics, where the fittest individuals are selected for reproduction to produce the offspring of the next generation. The process begins with the creation of an initial population, which is a set of randomly generated chromosomes. Each chromosome is evaluated for its fitness based on the performance of the model using those features. The best-performing chromosomes are then selected for reproduction through crossover and mutation to create a new population. This cycle repeats until a stopping condition is met. The parameter settings of n_population = 30, n_generations = 100, crossover probability = 0.8, and mutation probability = 0.2 were chosen to ensure sufficient exploration of the feature space while balancing computational efficiency. This process can be represented mathematically, including functions for fitness, crossover, and mutation, as detailed in the research work in [49].
- **FA**: This is inspired by the flashing behavior of fireflies. In FA, each firefly represents a potential solution, and the brightness of a firefly is determined by the objective function. Fireflies are attracted to each other based on their brightness, and the movement of a firefly is influenced by its attraction to brighter fireflies. The parameters n_fireflies = 30, max generations = 100, α=0.5, β=0.2, and γ=1.0 were selected to balance randomness. The details are outlined in [50].
- **WOA**: A nature-inspired optimization algorithm based on the social behavior of humpback whales. WOA mimics the bubble-net hunting strategy of whales, where they create bubble nets to encircle prey. In feature selection, each whale represents a potential solution, and the algorithm iterates through phases of encircling prey, bubble-net attacking, and searching for prey. The parameter settings of n whales = 30 and max iter = 100 were adopted to ensure consistent performance across datasets and sufficient iterations for convergence. Further details can be found in [51].

To ensure consistency of selected features across different training datasets in the 5-fold cross-validation protocol, feature selection was performed independently within each fold. This approach maintained a strict separation between training and testing data, preventing data leakage. Optimization algorithms (PSO, GA, FA, and WOA) were applied to the training subset in each fold to select features that maximized the F1.

The parameters for these algorithms were determined through an iterative process. The consistency of feature selection was assessed by recording the frequency of each feature being selected across all folds. Table 6 presents the features most consistently identified.

The feature selection process in this study was implemented using the Wrapper Feature Selection Toolbox (WFST) (https://github.com/JingweiToo/Wrapper-Feature-Selection-Toolbox-Python, (accessed on 1 January 2020)), which provides a comprehensive framework for applying various feature selection algorithms.

### 2.5. Machine Learning Algorithm and PSO Optimization

To classify fetal movement, we compared six ML algorithms, including RF [52], Support Vector Machines (SVMs) [53], k-Nearest Neighbors (kNNs) [54], NNs [55], Decision Trees (DTs) [56], and Extreme Gradient Boosting (XGB) [57] using scikit-learn toolkit (https://github.com/scikit-learn/scikit-learn, (accessed on 1 January 2020)). Each model underwent hyperparameter tuning with PSO to enhance performance. The PSO used the pyswarms toolkit (https://github.com/ljvmiranda921/pyswarms, (accessed on 1 January 2020)) with n_particles = 30, max_iter = 100. Combining scikit-learn with pyswarms allows for effective hyperparameter optimization, ensuring each ML model is finely tuned for optimal performance. Below are the details of the ML algorithm with the PSO tuning used:

- **RF** employs an ensemble of DT to improve classification performance and control overfitting. The selected best parameters by PSO optimization are n_estimators = 200, max_features = auto, max_depth = 4, and criterion = entropy.
- **SVM** aims to find a maximum-margin hyperplane in space to distinguish two types of samples. The selected best parameters by PSO optimization are C = 2, kernel = rbf, gamma = scale, and tol = 0.0001.
- **kNN** is an algorithm that finds the K instances closest to a new input in the training dataset. The best parameters selected by PSO are weights = distance, metric = manhattan, and n_neighbors = 3.
- **NN** is designed to capture complex patterns in data through multiple layers of interconnected neurons. The selected best parameters by PSO optimization are activation = relu, alpha = 0.0001, hidden_layer_sizes = (100, 50), learning_rate = constant, max_iter = 200, and solver = Adam.
- **DT** adopts the logic of if-then-else to construct a classifier with a tree structure. The selected best parameters by PSO are criterion = entropy, max_depth = None, min_samples_leaf = 1, min_samples_split = 2, and splitter = random.
- **XGB** is an implementation of gradient-boosted DT designed for speed and performance. The selected best parameters by PSO optimization are max_depth = 10, n_estimators = 60, and learning_rate = 0.1.

### 2.6. Performance Evaluation Criteria

To evaluate the performance of our classifiers, we employed k-fold cross-validation with K=5. This method involves splitting the dataset into five parts, where one part serves as the test subset while the remaining four parts are used for training. The performance metrics considered for this evaluation include SEN, PRE, and F1, which are defined as follows:

(2) $$\mathrm{Sensitivity}=\frac{TP}{TP+FN}$$

(3) $$\mathrm{Precision}=\frac{TP}{TP+FP}$$

(4) $$F1-\mathrm{score}=2\times \frac{\mathrm{Precision}\times \mathrm{Sensitivity}}{\mathrm{Precision}+\mathrm{Sensitivity}}$$

where a true positive (TP) means the model correctly detects fetal movement when the fetus is moving. A true negative (TN) means the model correctly identifies no movement when the fetus is not moving. A false positive (FP) occurs when the model incorrectly detects movement when the fetus is not moving, and a false negative (FN) occurs when the model fails to detect movement when the fetus is moving.

## 3. Results and Discussion

This section provides a detailed analysis of the performance of various classifiers in detecting fetal movements under different conditions. The performance of each classifier is evaluated using metrics including SEN, PRE, and F1. The results are reported as the mean values along with their variability, represented by the ± followed by the SD, derived from a 5-fold cross-validation process. Additionally, this section examines the system’s efficiency in data transmission and its performance in cloud storage.

### 3.1. Performance of Classifiers on Imbalanced Data Using Original Signals

Firstly, we conducted an extensive comparative analysis of six different models for detecting fetal movement. This section specifically focuses on evaluating these models using an imbalanced dataset to understand how effectively each one addresses class imbalance. The models utilized in this study include RF, SVM, kNN, NN, DT, and XGB.

The results presented in Table 4 reveal significant impacts of class imbalance on classifier performance, particularly evident in the varying behaviors across different models. The most notable effect is observed in the SVM classifier, where the combination of zero sensitivity 0.00 ± 0.00% and perfect precision 100.00 ± 0.00% demonstrates a severe bias toward the majority class. This behavior indicates that the model completely fails to learn minority class characteristics, defaulting to majority class predictions in all cases. Such results clearly illustrate how class imbalance can lead to models that appear accurate on surface-level metrics yet fail to provide meaningful predictions for the underrepresented class. When examining the F1, which represents the harmonic mean between sensitivity and precision, most models demonstrate poor performance. SVM achieves an F1 score of 0.00 ± 0.00%, reflecting a complete failure in minority class classification. Similarly, kNN shows a low F1 of 23.62 ± 4.23%, while NN performs slightly better with an F1 of 35.32 ± 5.93%. Decision Tree demonstrates moderate improvement with an F1 of 54.47 ± 7.09%. Both RF and XGB show the most promising results, with RF achieving an F1 score of 66.48 ± 11.98% and XGB exhibiting the best performance with an F1 of 74.81 ± 5.41%. This issue occurs because the datasets used in our study comprised 100 fetal movement data and 10,150 non-fetal movement data, resulting in a highly imbalanced distribution, with an imbalance ratio of 1:100. However, the generally low F1 scores across all models highlight the necessity for data balancing preprocessing, aiming to enhance the models’ ability to effectively classify both majority and minority classes [58]. To address this issue, the next section focuses on balancing the datasets to lessen the impact of class imbalance.

### 3.2. Performance of Classifiers Using SMOTE-Balanced Data

This section evaluates the performance of the models after implementing SMOTE. It was employed to balance the dataset by oversampling the minority class [59]. Importantly, It was applied strictly to the training dataset to prevent data leakage and maintain the integrity of the evaluation process on an unseen test dataset. The classification results using SMOTE-balanced data are presented in Table 5.

The results shown in Table 5 indicate that applying the SMOTE technique has improved the overall classification performance across all models. After implementing SMOTE, XGB achieved an F1 score of 77.92 ± 5.66%, RF reached 73.12 ± 5.37%, DT attained 59.17 ± 11.44%, NN obtained 53.56 ± 10.30%, kNN achieved 32.00 ± 4.01%, and SVM reached 18.64 ± 4.05%. These results demonstrate that the SMOTE technique successfully enhanced the performance of all classifiers compared to their initial F1 scores, indicating its effectiveness in addressing the class imbalance problem.

### 3.3. Performance of Classifier with Feature Selection

This subsection presents the classification performance of fetal movement detection using various feature selection methods, including PSO, GA, FA, and WOA. The specific positions of the selected features are detailed in Table 6 and the overall classification results for these methods are summarized in Table 7.

Table 6 demonstrates the effectiveness of feature selection algorithms such as PSO, GA, FA, and WOA in reducing feature complexity. These algorithms achieved a reduction in feature set size by approximately 43.33% to 50.00%, focusing on the most relevant features while discarding redundant or less informative ones. This capability not only simplifies the model but also aligns with prior research [60] that highlights the importance of dimensionality reduction in improving computational efficiency.

Furthermore, Table 7 reveals that the application of these feature selection methods led to improved classification performance compared to using the entire feature set. This improvement emphasizes the role of feature selection in enhancing model effectiveness by targeting relevant features. This finding is corroborated by the existing literature [61]. Among the evaluated methods, PSO outperformed GA, FA, and WOA in feature selection. The features selected by PSO include the Max of Ay, Az, Gy, and Gz; Min of Ay and Gz; Mean of Ax, Gx, and Gy; Median of Ax, Ay, Az, Gx, and Gy; Mode of Az, Gy, and Gz; SD of Ax, Az, Gx, Gy, and Gz; Var of Ax and Gy; Skew of Ay and Gx; Kurt of Ax; and CV of Ay, Gx, and Gz. With these optimized features, the PSO-XGB classifier achieved the highest classification performance, demonstrating a sensitivity of 88.00 ± 5.10, precision of 76.44 ± 7.35, and an F1-score of 81.61 ± 4.96. The superior performance of PSO can be attributed to its ability to effectively balance exploration and exploitation, allowing it to efficiently search the feature space and identify the most informative features [62].

Based on these findings, PSO-based ML models (PSO-RF, PSO-SVM, PSO-kNN, PSO-NN, PSO-DT, and PSO-XGB) have been selected as the standard models for further enhancement through hyperparameter tuning. This approach will be utilized in the next sections of our study.

### 3.4. Optimizing Performance with PSO Fine-Tuning

This section reports on the enhancement of PSO-based ML models through PSO for model fine-tuning. The results are presented in Table 8.

As shown in Table 8, PSO enhances the performance of all PSO-based ML models. This improvement is due to the optimization process and its ability to fine-tune model parameters, resulting in better convergence and more accurate classification outcomes as suggested in [63]. Notably, the PSO-based XGB classifier achieves the highest performance metrics. This performance is likely due to the inherent capabilities of XGB combined with the efficient parameter optimization provided by PSO.

**Figure 6:**
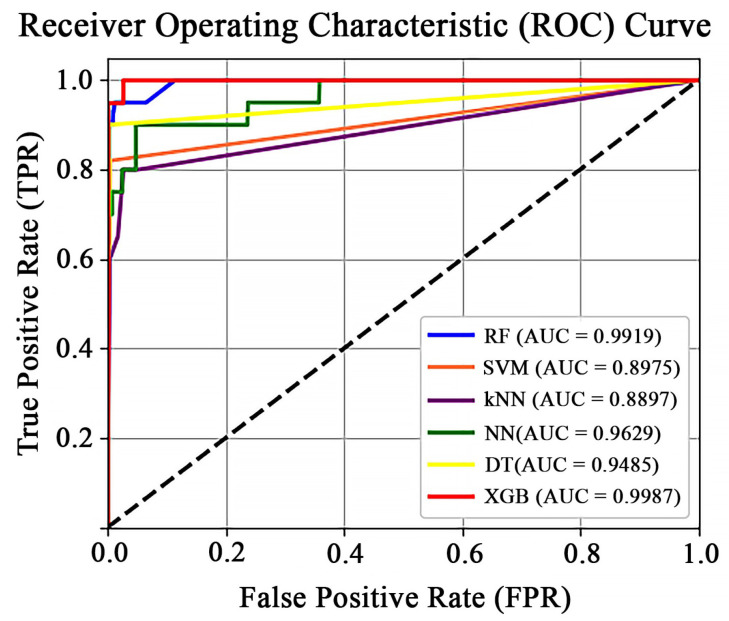
ROC curve comparison for various classification models.

In addition to SEN, PRE, and F1, we used the Receiver Operating Characteristic (ROC) curve to provide a comprehensive view of the models’ ability to distinguish between classes at various threshold settings. The ROC curve is a powerful tool for evaluating the performance of classification models. It plots the false positive rate against the true positive rate under different decision criteria, offering insights into the trade-offs between SEN and SPEC. Figure 6 presents the ROC curve comparison for various classification models. The XGB classifier performed the best, achieving the highest Area Under the Concentration–time curve (AUC) of 0.9987, followed by the RF, NN, DT, SVM, and kNN classifiers with AUC values of 0.9919, 0.9629, 0.9485, 0.8975, and 0.8897, respectively. Consequently, the PSO-based XGB classifier with PSO is embedded in our proposed device.

After evaluating the performance metrics of our proposed model, we conducted an additional analysis to compare our system with existing fetal movement detection systems. As seen in Table 9, several types of sensors such as piezoelectric, accelerometer, and EMG have been employed for fetal movement detection. Notably, fetal movement detection using accelerometer-based sensors tends to outperform those using another sensor. This is likely because accelerometers are more sensitive to fetal movements and robust enough to be ideal for long-term monitoring [64], whereas another sensor may be more affected by noise and signal inconsistencies.

Furthermore, ref. [40] demonstrates that combining accelerometers with piezoelectric and acoustic sensors enhances detection performance compared to using accelerometers alone. This improvement arises from the complementary detection capabilities of the different sensor types: accelerometers effectively capture linear movements, piezoelectric sensors detect pressure or strain changes, and acoustic sensors pick up sound and vibration signals. By integrating these diverse sensing modalities, the system can more accurately and reliably detect fetal movements, thereby enhancing overall performance. A similar pattern is observed in [31] and is reflected in our proposed device. In our proposed system, we demonstrate that incorporating both accelerometer and gyroscope sensors within a single device yields promising results. We employed PSO feature selection techniques to extract and retain relevant feature information from both sensors, effectively capturing essential data while reducing the dimensionality of the sensor outputs. For classification, we utilized an XGB classifier, known for high performance in handling structured data. The gyroscope complements the accelerometer by providing rotational movement data, allowing for a more comprehensive capture of fetal activity. Our system achieves a SEN of 90.00%, PRE of 87.46%, and F1 of 88.56%.

### 3.5. Data Transmission and Cloud Storage Performance

Data transmission and cloud storage performance are critical for the functionality of embedded wearable devices. Efficient data transmission is essential for continuous monitoring, impacting healthcare outcomes [65]. Our study evaluated the performance of the device over 100 cycles.

The results of our study on data transmission and cloud storage performance of the embedded wearable device are presented in Table 10. The latency in data transmission from the device to the cloud storage was observed to be 423.6 ms, which is the average with an SD of ±193.05 ms. Despite this, the data integrity and transmission success rate remained at 100%, ensuring that all data transmitted were accurately received and stored without any loss or corruption. Moreover, the device is capable of sustaining continuous operation for up to 48 h on a single charge. This battery performance is crucial for monitoring fetal movements over prolonged periods without requiring frequent recharging, thereby enhancing the usability and convenience for expectant mothers.

## 4. Conclusions and Future Work

In this study, we aimed to develop a wearable device for monitoring fetal movement both in and out of the hospital. Our proposed device integrates accelerometer and gyroscope sensors with IoT technology. Signals from these sensors were extracted using ten methods to serve as inputs for classification. We compared and analyzed six different ML algorithms and investigated four feature selection techniques to further improve performance. Experimental results demonstrated that our wearable device, utilizing PSO feature selection-based XGB with PSO hyper-tuning, achieved an SEN of 90.00%, PRE of 87.46%, and an F1 of 88.56%. Therefore, our proposed device incorporates the PSO-based XGB classifier with PSO. Incorporating IoT technology allows for continuous monitoring and data transmission to cloud-based systems. Over 100 cycles, the device demonstrated an average latency of 423.6 ms with an SD of 193.05 ms and maintained 100% data integrity and transmission success. The system was capable of operating continuously for up to 48 h on a single charge. This device provides an effective solution for detecting fetal movements, further enhancing maternal and fetal health monitoring. Additionally, all 35 volunteers participated in a post-test questionnaire using a five-point scale, with the majority of participants indicating a high level of comfort and convenience during device usage.

For our future work, we plan to utilize advanced classifiers and score combination methods to enhance classification performance [66,67] and integrate DNNs into the device. Additionally, we will expand our participant group to gather a larger dataset of fetal movement information [68]. Moreover, we plan to incorporate walking and other activity data into our training dataset to enhance our model’s ability to handle interference from maternal walking and position changes. We will also conduct in-depth power profiling and explore energy-efficient model architectures to optimize performance while minimizing power consumption. Furthermore, we aim to develop an automatic fetal movement monitoring and alert system, enabling early warnings for potential complications. Most importantly, we will conduct clinical case studies to evaluate the system’s effectiveness in real-world prenatal care settings.

## Figures and Tables

**Table 2:** Information of 35 pregnant women participants.

Participant No.	Age (Years)	Gestational Age ^1^	Weight (kg)	Abdominal Circumference (cm)	Fetal Position ^2^	Number of Fetal Movement
1	43	33 + 1	72.8	107	LOA	3
2	31	33 + 5	94	119	LOA	4
3	40	35 + 2	75.2	113	LOA	3
4	28	30 + 2	81	110	ROA	2
5	29	33 + 3	78	113	ROA	3
6	25	36 + 6	61	105	ROA	2
7	27	39 + 1	72.4	105	LOA	1
8	28	36	73	105	LOA	2
9	33	37 + 3	78.2	103	ROA	2
10	31	38 + 2	91.7	119	ROA	3
11	26	38	65	100	LOA	4
12	27	33 + 2	86.4	107	LOA	2
13	28	37 + 1	62.7	101	ROA	3
14	28	26 + 5	74.4	102	LOA	3
15	36	34 + 6	71	105	ROA	2
16	34	39	80.5	102	ROA	4
17	25	39	66	100	LOA	3
18	29	36 + 4	76.2	112	LOA	2
19	16	38 + 1	56.4	102	LOA	3
20	30	39 + 2	63.1	102	LOA	4
21	16	40 + 2	56.6	102	LOA	5
22	30	37 + 1	69	106	LOA	3
23	28	35 + 6	73.2	113	ROA	4
24	29	37 + 2	56.5	101	ROA	3
25	36	38 + 1	74.7	105	ROA	4
26	31	34 + 6	63.3	111	LOA	2
27	30	36 + 2	117.2	128	ROA	4
28	23	38 + 3	106	94	LOA	2
29	23	37 + 1	95	93	ROA	4
30	19	28 + 3	90	101	LOA	3
31	31	37 + 5	69.1	109	LOA	2
32	20	38	73.1	103	LOA	2
33	25	39	65.4	104	LOA	3
34	27	36 + 6	71.5	108	LOA	2
35	29	38 + 4	66.8	110	LOA	2

^1^ Weeks + day(s); ^2^ ROA: right occiput anterior, LOA: left occiput anterior.

**Table 3:** Features used in fetal movement detection.

Feature	Definition
Max	The highest recorded value of the segment.
Min	The lowest recorded value of the segment.
Mean	The average value of the segment.
Median	The middle value in the dataset of the segment.
Mode	The most frequently occurring value in the dataset of the segment.
SD	A measure of the amount of variation or dispersion of a set of values.
Var	The square of the SD.
Skew	A measure of the asymmetry of the probability distribution of the segment.
Kurt	A measure of the tailedness of the probability distribution of the segment.
CV	Measure of relative variability, expressed as the ratio of the SD to the mean, and is used to compare the dispersion between different signal.

**Table 4:** Performance of classifiers using original data.

Performance	RF	SVM	kNN	NN	DT	XGB
SEN (%)	56.00 ± 13.56	0.00 ± 0.00	35.00 ± 7.07	26.00 ± 4.90	55.00 ± 8.94	83.00 ± 6.00
PRE (%)	84.25 ± 5.68	100.00 ± 0.00	18.06 ± 3.54	55.73 ± 9.32	54.09 ± 5.27	68.16 ± 5.35
F1 (%)	66.48 ± 11.98	0.00 ± 0.00	23.62 ± 4.23	35.32 ± 5.93	54.47 ± 7.09	74.81 ± 5.41

**Table 5:** Performance of classifiers after implementing SMOTE.

Performance	RF	SVM	kNN	NN	DT	XGB
SEN (%)	73.00 ± 9.27	45.00 ± 6.32	60.00 ± 5.48	60.00 ± 14.49	54.00 ± 11.58	69.00 ± 5.83
PRE (%)	73.68 ± 3.08	12.05 ± 3.18	21.98 ± 3.56	50.03 ± 10.98	66.07 ± 11.74	89.72 ± 6.59
F1 (%)	73.12 ± 5.37	18.64 ± 4.05	32.00 ± 4.01	53.56 ± 10.30	59.17 ± 11.44	77.92 ± 5.66

**Table 6:** Positions of selected features for classification.

Feature Selection Method	Selected Features	Number of Features	Discarded Features	Feature Complexity Reduction (%)
PSO	$F_{1,2}$, $F_{1,3}$, $F_{1,5}$, $F_{1,6}$, $F_{2,2}$, $F_{2,6}$, $F_{3,1}$, $F_{3,4}$, $F_{3,5}$, $F_{4,1}$, $F_{4,2}$, $F_{4,3}$, $F_{4,4}$, $F_{4,5}$, $F_{5,3}$, $F_{5,5}$, $F_{5,6}$, $F_{6,1}$, $F_{6,3}$, $F_{6,4}$, $F_{6,5}$, $F_{6,6}$, $F_{7,1}$, $F_{7,5}$, $F_{8,2}$, $F_{8,4}$, $F_{9,1}$, $F_{10,2}$, $F_{10,4}$, $F_{10,6}$	30	30	50.00
GA	$F_{1,1}$, $F_{1,2}$, $F_{1,3}$, $F_{1,4}$, $F_{1,5}$, $F_{1,6}$, $F_{2,1}$, $F_{2,2}$, $F_{2,3}$, $F_{2,4}$, $F_{2,5}$, $F_{2,6}$, $F_{3,1}$, $F_{3,3}$, $F_{3,5}$, $F_{4,1}$, $F_{4,4}$, $F_{4,5}$, $F_{5,1}$, $F_{5,4}$, $F_{5,6}$, $F_{6,1}$, $F_{6,2}$, $F_{6,3}$, $F_{6,5}$, $F_{7,4}$, $F_{8,2}$, $F_{8,3}$, $F_{8,4}$, $F_{9,6}$, $F_{10,2}$, $F_{10,6}$	32	28	46.67
FA	$F_{1,2}$, $F_{1,3}$, $F_{1,4}$, $F_{1,5}$, $F_{1,6}$, $F_{2,1}$, $F_{2,3}$, $F_{2,6}$, $F_{3,2}$, $F_{3,4}$, $F_{3,6}$, $F_{4,1}$, $F_{4,2}$, $F_{4,3}$, $F_{4,4}$, $F_{5,2}$, $F_{5,4}$, $F_{5,5}$, $F_{6,2}$, $F_{6,4}$, $F_{6,5}$, $F_{6,6}$, $F_{7,1}$, $F_{7,2}$, $F_{7,5}$, $F_{7,6}$, $F_{8,3}$, $F_{8,5}$, $F_{9,1}$, $F_{9,3}$, $F_{9,6}$, $F_{10,2}$, $F_{10,4}$, $F_{10,6}$	34	26	43.33
WOA	$F_{1,2}$, $F_{1,3}$, $F_{1,4}$, $F_{1,5}$, $F_{1,6}$, $F_{2,1}$, $F_{2,3}$, $F_{2,4}$, $F_{3,2}$, $F_{3,3}$, $F_{3,5}$, $F_{4,1}$, $F_{4,2}$, $F_{4,3}$, $F_{4,4}$, $F_{5,2}$, $F_{5,4}$, $F_{5,5}$, $F_{6,2}$, $F_{6,4}$, $F_{6,5}$, $F_{6,6}$, $F_{7,1}$, $F_{7,2}$, $F_{7,5}$, $F_{7,6}$, $F_{8,2}$, $F_{8,5}$, $F_{9,1}$, $F_{9,3}$, $F_{9,6}$, $F_{10,2}$, $F_{10,4}$, $F_{10,6}$	34	26	43.33

**Table 7:** Performance of classifiers using feature selection methods.

Feature Selection Method	Classifier	SEN (%)	PRE (%)	F1 (%)
	RF	84.00 ± 5.83	76.88 ± 9.04	80.20 ± 7.43
	SVM	62.00 ± 5.10	53.85 ± 8.43	57.27 ± 6.12
PSO	kNN	34.00 ± 2.00	86.67 ± 10.88	48.56 ± 1.84
	NN	67.00 ± 2.45	69.43 ± 5.16	68.09 ± 2.91
	DT	76.00 ± 8.00	66.46 ± 8.05	70.78 ± 7.53
	XGB	88.00 ± 5.10	76.44 ± 7.35	81.61 ± 4.96
	RF	80.00 ± 5.48	75.05 ± 5.08	77.30 ± 4.10
	SVM	65.00 ± 9.49	46.72 ± 5.54	54.11 ± 5.95
GA	kNN	37.00 ± 2.45	66.98 ± 11.66	47.30 ± 3.88
	NN	72.00 ± 10.77	64.59 ± 6.90	67.76 ± 7.74
	DT	73.00 ± 7.48	53.30 ± 4.36	61.35 ± 3.98
	XGB	86.00 ± 6.63	73.53 ± 4.86	79.23 ± 5.25
	RF	81.00 ± 5.83	76.40 ± 8.41	78.41 ± 6.14
	SVM	61.00 ± 11.58	48.81 ± 2.74	53.79 ± 5.17
FA	kNN	34.00 ± 2.00	81.31 ± 4.36	47.85 ± 1.83
	NN	62.00 ± 12.08	60.33 ± 10.29	60.40 ± 0.18
	DT	72.00 ± 17.49	52.85 ± 5.40	60.44 ± 9.28
	XGB	86.00 ± 8.00	74.88 ± 3.40	79.87 ± 4.30
	RF	81.00 ± 8.60	72.54 ± 10.70	76.27 ± 8.88
	SVM	63.00 ± 6.78	50.27 ± 3.07	55.88 ± 4.52
WOA	kNN	36.00 ± 3.74	53.49 ± 10.28	42.86 ± 5.81
	NN	66.00 ± 10.19	61.21 ± 6.23	62.68 ± 3.63
	DT	73.00 ± 14.35	54.79 ± 5.42	62.17 ± 8.34
	XGB	82.00 ± 8.72	75.04 ± 6.69	78.05 ± 5.69

**Table 8:** Performance of classifiers after PSO feature selection-based optimization.

Performance	PSO-RF	PSO-SVM	PSO-kNN	PSO-NN	PSO-DT	PSO-XGB
SEN (%)	85.00 ± 7.75	73.00 ± 5.10	36.00 ± 3.74	67.00 ± 9.27	80.00 ± 8.36	90.00 ± 7.74
PRE (%)	87.73 ± 5.82	65.37 ± 7.83	94.64 ± 6.58	79.26 ± 7.90	76.95 ± 5.40	87.46 ± 4.58
F1 (%)	86.20 ± 6.11	68.74 ± 5.60	52.11 ± 4.77	71.78 ± 4.90	78.30 ± 6.05	88.56 ± 7.28

**Table 9:** Comparison of model performance.

Reference	Sensor	Performance
Mesbah et al. (2011) [29]	Four accelerometers	SEN = 76.00%, SPEC = 55.00%, F1 = 0.59
Altini et al. (2016) [30]	Six accelerometers	SEN = 75.00%
Altini et al. (2017) [31]	Six accelerometers and one EMG	SEN = 74.00%
Abeywardhana et al. (2018) [32]	One accelerometer	ACC = 95.00%
Zhao et al. (2019) [28]	Four accelerometers	N/A
Xu et al. (2022) [36]	Two accelerometers	SEN = 82.40%, PRE = 86.10%,
		F1 = 84.20%, ACC = 86.60%
Qin et al. (2023) [27]	Two accelerometers	ACC = 89.74%
Ghosh et al. (2024) [39]	Two accelerometers	ACC = 89.74%
Ghosh et al. (2024) [40]	One accelerometer and one acoustic	SEN = 73.00%, PRE = 72.00%, F1 = 73.00%
	One accelerometer and one piezoelectric	SEN = 79.00%, PRE = 71.00%, F1 = 74.00%
	One acoustic and one piezoelectric	SEN = 81.00%, PRE = 75.00%, F1 = 78.00%
	One accelerometer, one piezoelectric and one acoustic	SEN = 82.00%, PRE = 76.00%, F1 = 79.00%
**This work**	**One accelerometer and one gyroscope**	**SEN = 90.00%, PRE = 87.46%, F1 = 88.56%**

**Table 10:** Data transmission and cloud storage performance.

Metric	Value
Latency	423.6 ± 193.05 ms
Data Integrity	100%
Transmission Success Rate	100%
Battery Life	48 h

## Data Availability

Data are contained within the article.
